# Next generation sequencing identified novel heterozygous nonsense mutation in *CNGB1* gene associated with retinitis pigmentosa in a Chinese patient

**DOI:** 10.18632/oncotarget.21728

**Published:** 2017-10-10

**Authors:** Santasree Banerjee, Junping Yao, Xinxin Zhang, Jianjun Niu, Zhongshan Chen

**Affiliations:** ^1^ Department of Cell Biology and Medical Genetics, School of Medicine, Zhejiang University, Hangzhou, China; ^2^ Department of Ophthalmology, Tianyou Hospital Affiliated to Wuhan University of Science & Technology, Wuhan, China; ^3^ Department of Ophthalmology, General Hospital of Xinjiang Military Area Command of Chinese PLA, Urumqi, China; ^4^ Department of Ophthalmology, Zhongnan Hospital of Wuhan University, Wuhan, China

**Keywords:** retinitis pigmentosa, CNGB1gene, midperipheral visual field, rod photoreceptor cells, loss of vision, Pathology Section

## Abstract

Retinitis pigmentosa (RP) is a severe hereditary eye disease characterized by progressive degeneration of photoreceptors and subsequent loss of vision. Retinitis pigmentosa (RP) is a clinically and genetically heterogeneous group of retinal diseases. Germline mutations of *CNGB1* is associated with retinitis pigmentosa. We have identified and investigated a 34-year-old Chinese man with markedly have night vision blindness and loss of midperipheral visual field. The proband also lose his far peripheral visual field and also central vision. Proband’s retinal pigment deposits visible on fundus examination and primary loss of rod photoreceptor cells followed by secondary loss of cone photoreceptors. Target exome capture based next generation sequencing and Sanger sequencing identified novel nonsense mutation, c.1917G>A and a reported mutation, c.2361C>A, in the *CNGB1* gene. Both the nonsense mutations are predicted to lead to the formation of a premature stop codon which finally results into formation of truncated CNGB1 protein product which finally predicted to be disease causing. According to the variant classification guidelines of ACMG, these two variants are categorized as “*likely pathogenic*” variants. Our findings expand the mutational spectra of *CNGB1* and are valuable in the mutation-based pre- and post-natal screening and genetic diagnosis for retinitis pigmentosa.

## INTRODUCTION

Retinitis pigmentosa (RP) is an inherited eye disease with dystrophy of retina dystrophy that finally results into loss of vision. It is genetically and phenotypically extremely heterogenous. According to inheritance pattern, disease onset and clinical symptoms, retinitis pigmentosa is showing extreme heterogeneity. There are almost one and half a million patients with RP, around the world [1]. Patients with RP experienced with degradation of retinal cone and rod cells which finally causes blindness [2]. According to the mode of inheritance, RP has been inherited either dominantly or recessively or with a X-linked pattern [3]. However, very rare cases it has been showed that RP is caused by bigenic pattern. In addition, half of the RP cases are inherited either dominantly (20%) or recessively (20%) or with a X-linked (10%) pattern [4-5]. Due to extreme genetic heterogeneity, genetic screening for RP patients and identifying the causal variants from candidate genes are really a big challenge [6-7].

Based on classical genetics, recessively inherited disease majorly caused by homozygous mutation of a candidate gene in consanguineous families [8, 9]. For such cases, homozygosity mapping is a significant technology for identifying the responsible genes [10]. So, for identifying compound heterozygosity, target exome capture based next generation sequencing technology is the most efficient.

Here, in order to identify the molecular basis of RP in the proband of this Chinese family, we screened a panel of 60 genes associated with retinitis pigmentosa by targeted next-generation sequencing and confirmatory direct sequencing. In this study, we found novel heterozygous nonsense mutations of *CNGB1* gene segregating with retinitis pigmentosa phenotype in the proband, with autosomal recessive (compound heterozygosity) inheritance.

## RESULTS

### Clinical description

**Figure 1:**
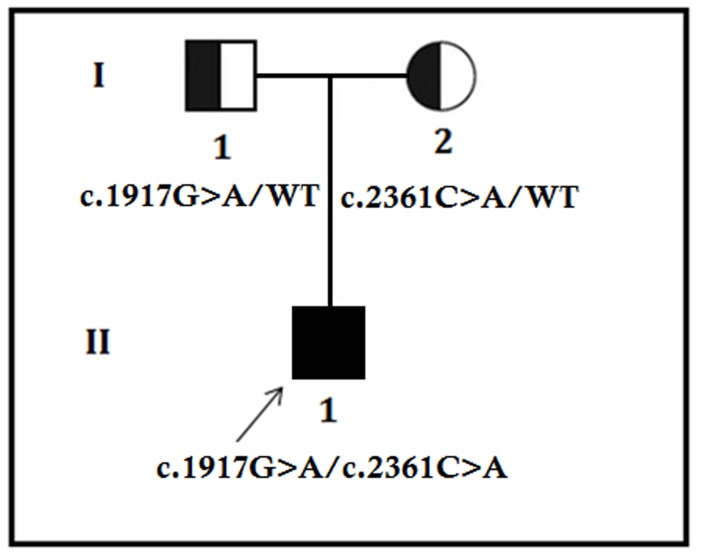
Pedigree of the family The filled symbol indicates the patient (proband), and the half-filled symbols show the carrier parents, who were heterozygous carriers but were unaffected. The arrow points to the proband.

A 28 years old Chinese man of a non-consanguineous Chinese family with RP was studied. The pedigree contained only one affected individual (The proband, II-1) (Figure 1). Since childhood, the proband (II-1) has been presented with night blindness with consecutive loss of peripheral vision. During ophthalmic examination (28 years), spicule-shaped pigmentory deposits has been identified in the fundus with progressive reduction of the visual field in both the eyes.

All the family members have given their informed consent for participating in this study.

### Identification and characterization of candidate mutation

**Figure 2:**
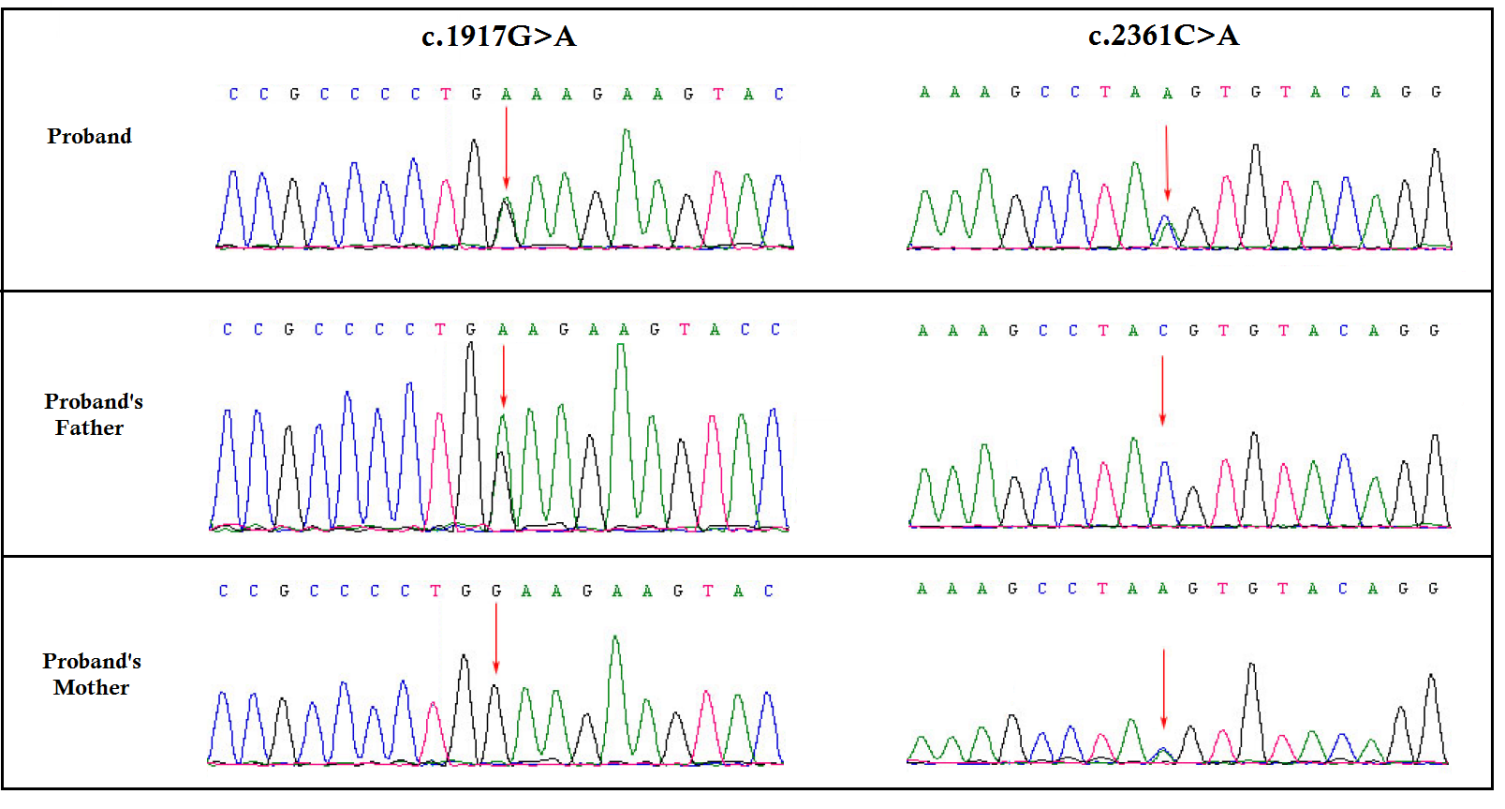
Partial DNA sequences in the *CNGB1* by Sanger sequencing of the family [NM_001297] Upper line: the proband, middle line: the father, bottom line: the mother. Arrows point to the mutations. The proband inherited both c.1917G>A and c.2361C>A mutations. The father carries the c.1917G>A mutation, and the mother carries the c.2361C>A mutation.

We identified two novel nonsense mutation in *CNGB1*: c.1917G>A in exon 20, and c.2361C>A in exon 24, inherited from the healthy father and mother respectively (Figure 2). Both of these mutations are predicted to lead to the formation of truncated CNGB1 protein. c.1917G>A and c.2361C>A leads to the formation of a premature sop codon which in turn predicted to results in premature termination of translation p.Trp639* and p.Tyr787* respectively. In addition, *in silico* analysis showed these two novel mutations are potential to cause disease [11, 12]. These two mutations are classified as “likely pathogenic” variant based on ACMG guidelines [13].

We did not detect these two mutations in the 100 normal control individuals of the same ethnic origin, gender and age range.

## DISCUSSION

In our study, we found two heterozygous *loss-of-function* mutations (c.1917G>A, p.Trp639*; and c.2361C>A, p.Tyr787*) [NCBI Reference sequence NM_001297] of the human *CNGB1* gene in the proband (II-1). Both of these heterozygous nonsense mutations of *CNGB1* gene are predicted to form truncated CNGB1 protein in contrast with the wildtype CNGB1 protein consisting of 1251 amino acids. c.2361C>A, p.Tyr787* was previously reported by Xu et al., in 2014 [14].

Germline mutations in *CNGB1* genes is associated with autosomal recessive retinitis pigmentosa (arRP) very rare. Clinical manifestations of our patient are same with all RP patients with mutations *CNGB1* gene reported previously.

CNGB1 encode β-subunits of cyclic nucleotide-gated (CNG) channels plays a significant role for signal transduction pathways in both visual and olfactory system. These are ligand-gated channels regulated or controlled directly by second messenger like Cyclic guanosine monophosphate or cyclic adenosine monophosphate. CNG channels control the photo-transduction pathways in photoreceptor cells which is finally leads to photoreceptor cell hyperpolarization [15]. However, CNG channels are composed by a heterotetramer including an α-subunit dimer and a β-subunit dimer [16-19]. Additionally, structurally and functionally α- and β-subunits are sharing close similarity [20].

Moreover, wild type α-subunit and β-subunit are required for normal function of CNG channel. Truncating mutation in *CNGB1* gene causes formation of non-functional CNGB1 protein which finally affects the normal function of the CNG channel [21, 22].

In conclusion, here, we report a Chinese patient who presented with retinitis pigmentosa, with novel mutations in the *CNGB1* gene. Our study is significant for genetic screening and clinical diagnosis of retinitis pigmentosa.

## MATERIALS AND METHODS

### Ethical statement

Proband and his parents of this Chinese family have given written informed consent as they are participating in this study. The Ethical Committee of the Department of Ophthalmology, Zhongnan Hospital of Wuhan University, Wuhan, China, reviewed and approved our study protocol in compliance with the Helsinki declaration. Diagnosis of the patients for retinitis pigmentosa has done by ophthalmologist.

### Patients and pedigree

A proband of Chinese descend with retinitis pigmentosa, diagnosed in the Department of Ophthalmology, Zhongnan Hospital of Wuhan University, Wuhan, China, were enrolled in our study.

### Targeted exome-based next-generation sequencing and variant identification

DNA samples obtained from the proband (II-1) were sequenced using target exome-based next-generation sequencing. Roche NimbleGen’s (Madison,USA) custom Sequence Capture Human Array was used to designed to capture 221340 kb of targeted sequence, covering 181 exons and flanking sequence (including the 100 bp of introns) of 60 genes (*ABCA4, AIPL1, ARL6, BEST1, C2orf71, CA4, CDHR1, CERKL, CLRN1, CNGA1, CNGB1, CRB1, CRX, CYP4V2, DHDDS, EYS, FAM161A, FLVCR1, ,FSCN2, GUCA1B, IDH3B, IMPDH1, IMPG2, KLHL7, LRAT, MAK, MERTK, NR2E3, NRL, OFD1, PDE6A, PDE6B, PDE6G, PRCD, PROM1, PRPF3, PRPF6, PRPF8, PRPF31, PRPH2, RBP3, RDH12, RGR, RHO, RLBP1, ROM1, RP1, RP2, ,RP9, RPE65, RPGR, SAG, SEMA4A, SNRNP200, SPATA7, TOPORS, TTC8, TULP1, USH2A, ZNF513*) associated with retinitis pigmentosa and yielded an average of 6366534 reads per sample, with approximately 96.92% mapping to the targeted regions. The average sequencing depth of the target area is 217.08% with 99.73% coverage. The procedure for preparation of libraries was consistent with standard operating protocols. In each pooling batch, 10 to 33 samples were sequenced simultaneously on Illumina HiSeq 2500 Analyzers (Illumina, San Diego, USA) for 90 cycles (specially designed by us for this study). Image analysis, error estimation, and base calling were performed using Illumina Pipeline software (version 1.3.4) to generate raw data. The raw reads were screened to generate – clean reads‖ followed by established filtering criteria. Clean reads with a length of 90 bp were aligned to the reference human genome from the NCBI database (Build 37) using the Burrows Wheeler Aligner (BWA) Multi-Vision software package with output files in - bam‖ format. The bamdata were used for reads coverage in the target region and sequencing depth computation, SNP and INDEL calling, and CNV detection. First, a novel three-step computational frame work for CNV was applied. Then, SNPs and INDELs were called using SOAPsnp software and Sam tools pileup software, respectively. A SNP or INDEL was be filtered if it could not follow the criterion: supported by at least 10 reads and >20% of the total reads. The frequency filter was set at 0.05. If a SNP frequency was more than 0.05 in any of the four databases (dbSNP, Hapmap, 1000 Genomes Project, the 124 healthy reference samples sequenced in this study), it would be regarded as a polymorphism, but not a causative mutation.

**Figure 3:**
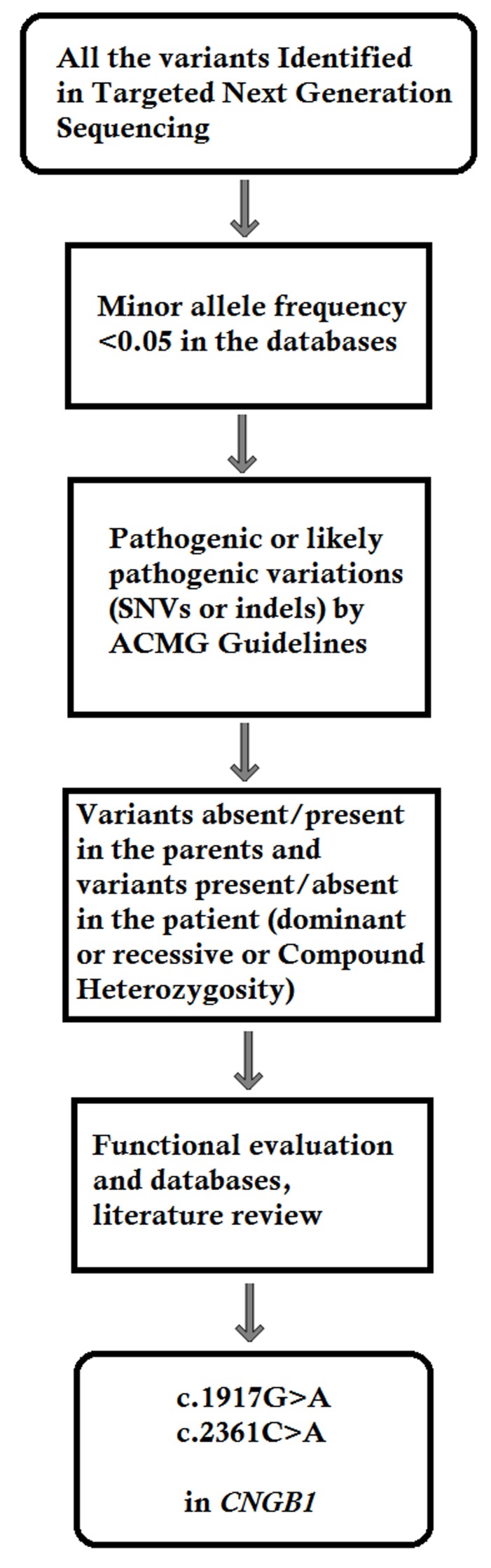
Filtering process for pathogenic mutations in all variations obtained by exome sequencing *Databases used: dbSNP, Hapmap, 1000 Genomes Project and BGI’s in–house database of ∼30000 Chinese people. SNV: single nucleotide variation. Indel: small insertion and deletion.

Last, SNVs were retrieved in The Human Gene Mutation Database (http://www.hgmd.cf.ac.uk/ac/index.php) and the Leiden Open Variation Database (http://www.lovd.nl/3.0/home), and then labeled as reported or novel (Figure 3).

### Confirmation of the novel splice-site mutation by Sanger sequence

To validate true positive of the mutation, Sanger sequencing was performed. Primers flanking the candidate loci were designed based on the reference genomic sequences of Human Genome from GenBank in NCBI and synthesized by Invitrogen, Shanghai, China. PCR amplification was carried out in ABI 9700 Thermal Cycler. PCR products were directly sequenced on ABI PRISM 3730 automated sequencer (Applied Biosystems, Foster City, CA, USA). Sequence data comparisons and analysis were performed by DNASTAR SeqMan (DNASTAR, Madison, Wisconsin, USA).

These two heterozygous novel nonsense mutations identified through targeted next generation sequencing were verified through Sanger sequencing using the primers: F1 5’-TTTACCAGTGAGGGACGGGC-3’, R1 5’-GTTTGTCTGGCTCCGGTAAGTA-3’. The reference sequence NM_001297 of *CNGB1* was used.
